# Transient Complete Resolution of Tourette Syndrome Symptoms Following Personalized Depth Electrode Placement

**DOI:** 10.3390/brainsci11121559

**Published:** 2021-11-25

**Authors:** Jennifer A. MacLean, Diana Ferman, Jason K. Chu, Mark A. Liker, Terence D. Sanger

**Affiliations:** 1 Department of Neurology, Children’s Health of Orange County, Orange, CA 92868, USA; jmalean@choc.org; 2 Department of Neurology, University of Southern California, Los Angeles, CA 90033, USA; dferman@usc.edu; 3 Department of Neurosurgery, University of Southern California, Los Angeles, CA 90033, USA; jachu@chla.usc.edu (J.K.C.); liker@usc.edu (M.A.L.); 4 Department of Neurosurgery, Children’s Hospital of Los Angeles, Los Angeles, CA 90027, USA; 5 Department of Electrical Engineering and Computer Science, University of California, Irvine, CA 92697, USA

**Keywords:** Tourette syndrome, deep brain stimulation, pediatric, targeting, stereo EEG

## Abstract

Treatment refractory Tourette syndrome has been shown to be improved with deep brain stimulation, but with multiple possible stimulation locations and variable and incomplete benefit. This study presents a single case of complete amelioration of motor and verbal tics in a patient with Tourette syndrome during placement of 12 stereo-EEG electrodes to identify optimal targets for permanent stimulating electrodes. Subsequently, substantial improvement in motor and verbal tic frequency occurred with placement and programming of permanent electrodes in bilateral globus pallidus internus and nucleus accumbens, but without the complete resolution seen during depth electrode placement. We suggest that simultaneous stimulation at multiple patient-specific targets could provide effective control of Tourette symptomatology, but further study will be needed.

## 1. Introduction

Deep brain stimulation (DBS) has been used as a surgical treatment for severe medication-refractory Tourette syndrome for over 20 years [1]. DBS has been reported to decrease the severity of motor and vocal tics in patients with Tourette syndrome [2]. However, the optimal targets and stimulation settings remain unknown; some patients fail to respond to therapy, and most experience only incomplete benefits. Variable results could be due to the possibility that the optimal target for DBS varies between patients, even when symptoms are similar.

Multiple potential targets have been identified, including globus pallidus internus (GPi) anterior, GPi posterior, centromedian thalamus, nucleus accumbens (NA), and the anterior limb of the internal capsule [2,3]. The variability in target location among patients presents numerous potential areas for lead implantation to provide benefit, and may be patient-specific. Despite such adjustments, treatment may not be sufficient to achieve complete resolution of the patient’s symptoms [4], although in some cases excellent results have been reported [5], with benefit up to 1 year after surgery. The mechanism of efficacy in Tourette Syndrome is not known, although efficacy of targets that have been shown to be effective in other disorders such as Parkinson’s disease [6] and generalized dystonia [7] suggests that a correction of basal ganglia dysfunction or blocking of inappropriate signals in the basal ganglia–thalamo–cortical loops at various points could be partly responsible for benefit.

It is possible that different targets work by different mechanisms. For example, efficacy of nucleus accumbens stimulation in both Tourette syndrome and obsessive-compulsive disorder [8] suggests a similarity in the modulation of volitional or compulsive triggers of movement. We present a unique case of transient complete amelioration of motor and verbal tics in an adolescent patient with Tourette syndrome during DBS targeting. This case suggests that complete resolution could be possible with appropriate targeting, multiple stimulation sites, and optimized programming.

## 2. Materials and Methods

### 2.1. Case Presentation

A 15-year-old male patient presented to the neurology department at our institution with severe Tourette syndrome and comorbid anxiety and depression. The patient’s parents reported that tic onset had occurred when the patient was 9 years of age, when the patient began to exhibit a vocal tic (throat grunting). Motor tics developed at 11 years of age. The tics had worsened over the following three years, at which point the patient developed coprolalia. The patient presented to our movement disorders clinic with severe coprolalia and near-continuous simple and complex motor tics. The patient’s tics were noted to significantly limit his activities of daily living, including self-feeding, self-care, social interaction, and school attendance. Attempts to type were unsuccessful, resulting only in repeated hits to the keyboard interrupted by motor tics. At the time of the initial consultation, the patient had been unable to attend school for over 8 months. He ex-pressed significant feelings of isolation and previous suicidal ideation 6 months prior to consultation. The patient displayed occasional self-injurious tic behavior, including lip/tongue biting and hitting himself in the arm and/or chest. Coprolalia was suggestible and triggerable, with frequency greater than 1/min, concurrent with complex motor tics.

Multiple attempts at medical therapy included trials of escitalopram, benztropine, clonazepam, clonidine, sertraline, haloperidol, risperidone, guanfacine, and aripiprazole, all of which failed to decrease tic frequency for more than 4 weeks, at any dose administered. Escalation rate, maximum dose, duration, and concomitant medications were adjusted carefully to ensure that the failure of medical therapy was confirmed by an adequate medication trial. After treatment with a single dose of haloperidol, the patient had an acute dystonic reaction requiring hospitalization. Dystonia at that time was diagnosed by a pediatric movement disorder specialist (TDS) based on published criteria [9,10]. Haloperidol treatment was discontinued, and dystonia was resolved.

Given his lack of response to multiple medications and the severity of symptoms pre-venting activities of daily living including education, DBS surgery was discussed with the patient and his family. Since the optimal DBS target for Tourette syndrome is not known, we recommended use of a staged procedure with implantation of test leads at multiple possible targets [11]. The patient and his family consulted with neurosurgery (JKC, MAL) and elected to proceed with implantation of temporary depth electrodes at 12 sites and an anticipated hospitalization for testing of approximately 4–7 days. The presurgical work-up included neuroimaging and laboratory studies, which demonstrated no abnormalities or contraindications to surgical intervention. The patient’s mental health counselor expressed no contraindications. He was off all medications at the time of testing.

### 2.2. Deep Brain Stimulation

In this case, we used a previously reported DBS technique [11,12] based on the use of stereoelectroencephalography (sEEG) depth electrodes that are commonly used to identify epileptic foci [13]. Temporary depth electrodes are used for test stimulation and recording at multiple possible targets. The information obtained is used for the subsequent implantation of permanent DBS electrodes.

#### 2.2.1. Implantation

DBS is a reversible procedure, but precise target localization is critical to efficacy [14]. Potential targets for stimulation were identified by consultation with the Departments of Neurology and the Department of Neurosurgery at our institution, as well as a review of reports in the literature of efficacy in the treatment of dystonia and Tourette syndrome through either lesions or stimulation in thalamus and basal ganglia [2,3,15].

Stereotactic placement of temporary electrodes was initially performed to optimize final implant location. A preoperative stereotactic magnetic resonance imaging (MRI) scan with high resolution T1 and T2 sequences was performed. Targeting of the bilateral centromedian parafasicular nuclei of thalamus (CM), VIM thalamus, subthalamic nuclei (STN), GPi anterior, GPi posterior, and NA was completed on the ROSA ONE™ software (Zimmer Biomet, Montpellier, France). Targeting was based off standard surgical anatomy Schaltenbrand-Wahren atlas locations based on ACPC coordinates with adjustment based on the patient’s anatomy and is shown in Table 1.

After induction of general anesthesia, six bone fiducial markers (Medtronic Inc., Minneapolis, MN, USA) were secured to the skull and a stereotactic computed tomography (CT) scan was obtained. The CT scan was co-registered with the preoperative MRI. The patient was placed in the Mayfield clamp (Integra, Princeton, NJ, USA) and registered to the ROSA stereotactic robot with an accuracy of <0.5 mm.

**Figure 1:**
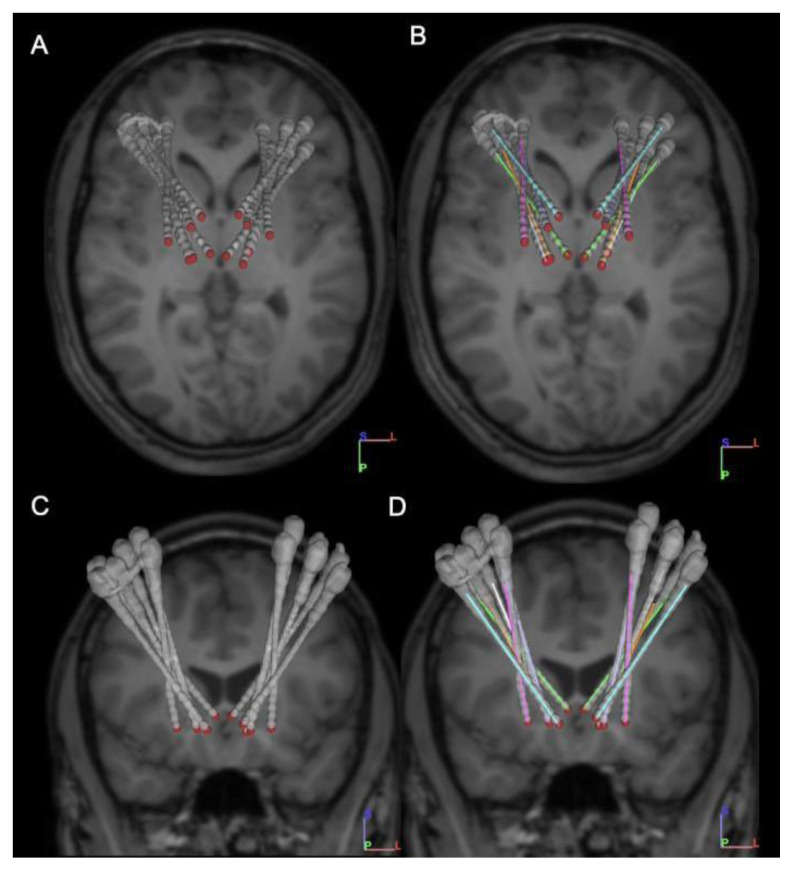
Axial (**A**,**B**) and coronal (**C**,**D**) views of the postoperative CT overlaid on the preoperative MRI, showing the lead locations for the Adtech stereo EEG leads.

Under ROSA stereotactic guidance, a total of 12 Adtech MM16C depth electrodes (Adtech Medical Instrument Corp., Oak Creek, WI, USA) were placed via drill holes in bilateral CM parafasicular nuclei of thalamus, VIM thalamus, Vo thalamus-STN, GPi anterior, GPi posterior, and NA. Each electrode has a diameter of 1.2 mm, and includes 6 low-impedance (1–2 kOhm) contacts that run circumferentially around the electrode in a 2 mm band suitable for stimulation. There is a 5 mm distance between the centers of each low impedance contact. Each electrode also contains 10 high-impedance (70–90 kOhm) contacts that are approximately 500 μm circular and arranged in groups of 2 or 3 circumferentially around the electrode both at the tip and between the low-impedance electrode bands, which are appropriate for recording of local field potentials (LFP). Electrodes were fixed to the skull through 13-mm anchor bolts (Adtech LSBK1-BX-06, Adtech Medical Instrument Corp., Oak Creek, WI, USA). Postoperative CT was performed to confirm accurate localization of the electrodes, and to rule out implantation related hemorrhage or other complications. Final electrode placement is shown in Figure 1.

#### 2.2.2. Test Stimulation

Test stimulation was performed in an inpatient “neuromodulation monitoring unit” (NMU). Depth Electrodes (Adtech MM16C) were maintained with the proximal end externalized and connected to external recording and stimulation equipment (WS8 high performance computer workstation, RZ2 bioamp processor, and RS4 streaming storage processor, Tucker-Davis Technologies Inc., Alachua, FL, USA) Custom unity-gain buffer amplifiers were embedded in the “cabrio” connectors attached to the depth electrodes. The patient’s head was wrapped in sterile gauze in order to protect the electrode entry points through the scalp. The patient was connected to the recording equipment through 2 m shielded cables. Other than limitations of the cable length, the patient was free to move about the bed and hospital room, and was able to ambulate when recording equipment could be moved with him.

In order to permit full recovery from the effects of general anesthesia, testing did not start until 24 h after surgery. Testing included three categories of data: (1) stimulation; (2) recording of spontaneous brain activity; and (3) evoked potentials in response to electrical stimulation. Initial testing involved LFP recordings during activities, including active and passive movement, followed by evoked potential assessment. Recordings were obtained from the MM16C high-impedance contacts (50 μm micro-wires, 80–90 kOhm) sampled at 22 kHz. Evoked potentials were recorded with the same parameters during stimulation and subsequently stimulus-locked averaged off-line. Video was recorded continuously and synchronized to the intracranial recordings using either an in-room camera or via USB-connected peripheral camera.

Stimulation was performed through pairs of low-impedance MM16C contacts (1–3 kOhm) after confirming the integrity of the leads by checking impedance. Stimulation and impedance testing are performed using the Medtronic external neurostimulator 37022 with a Medtronic 8840 DBS programmer (Medtronic Inc., Minneapolis, MN, USA) at bilateral (homologous left and right) contacts with settings of 90 μs, 60 Hz or 185 Hz, and increasing voltage up to 5 V, for a total of 3–5 min per condition. During stimulation, the patient was observed for beneficial effects, including reduction in frequency of tics and tic volume as well as any subjective improvements noted by patient. Clinically beneficial contacts were subsequently probed with both bilateral and unilateral assessment, with adjustments in frequency by 5 Hz steps and pulsewidth by 10 us steps to find optimal settings. Due to the need to test multiple contacts at multiple frequencies, testing required 1–2 days, with the patient speaking, moving, or otherwise performing activities during which tics would normally be evident. Once effective stimulation parameters were identified, testing was repeated twice 24 h apart to ensure consistent response. Blinded stimulation to the patient and an examiner was also performed (stimulation adjustments made by additional clinician) to confirm clinical efficacy.

#### 2.2.3. Perioperative Medication

The patient did not complain of discomfort with electrodes inserted, and transient peri operative pain was treated with acetaminophen. In order to maintain wakefulness and provoke tics for observation of stimulation effects, methylphenidate 5–10 mg orally was administered intermittently during hospitalization.

## 3. Results

### Outcome Measures

Upon awakening from electrode implantation, the patient was noted to have complete cessation of motor and vocal tics. Multiple attempts to elicit tics, including the use of intravenous steroids to minimize a possible inflammatory micro-lesion effect, stimulant medication, and environmental triggers, were unsuccessful. Only rare verbal tics without coprolalia or any clear pattern were noted to occur. Although a rare episode of coprolalia occurred on day 5 post-lead implantation, during a direct discussion regarding the patient’s tics and his emotional and physical stressors, coprolalia could not be otherwise elicited. At this time stimulation testing was initiated in order to evaluate for potential settings. Assessment of evoked potentials and local field potentials was repeated during this time. Tics began to appear with slightly increasing frequency on day 10 postoperatively, but still required specific elicitation such as emotional triggers for the patient. At no point did the tic severity return to the prior level, and coprolalia did not occur spontaneously during the hospitalization. Tics remained absent while the patient performed regular activities in the context of social interaction, family discussions, and playing video games. Notably, prior to surgery, the patient’s tics were so severe that they persisted even when the patient was alone and at rest, interfering with sleep onset. In order to evaluate the DBS effect on tics, the patient was hospitalized for approximately 2 weeks until tic frequency had increased to sufficient regularity for testing.

10 days after test lead implantation, stimulation was repeated to evaluate the effect on tic frequency. The greatest decrease in tic frequency was elicited with concurrent stimulation to bilateral GPi posterior and NA. Stimulation to CM parafascicular nuclei also had benefit, although slightly less. Optimal stimulation settings in GPi posterior (with 1 being the most distal contact) were 123-4+ at 3v, 120 μs, and 40 Hz bilateral. Optimal stimulation settings in left NA were 123-4+ at 5v, 120 μs, and 180 Hz. Optimal settings on right NA were 234-5+ at 5v, 120 μs, and 180 Hz. Externalized electrodes were removed 12 days after implantation. Worsening of motor tics was observed approximately 5 h after removal, with the progressive re-emergence of motor and vocal tics, including coprolalia, over subsequent days. The patient was discharged on post-operative day 12.

Recorded data from the MM16C micro-contacts showed variable activity in all recorded regions associated with voluntary movement. When tics recurred, widespread brief activity in NA and CMpf often occurred at the time of the tic, but not always preceding movement onset. Reliable analysis and presentation of the recorded data requires confirmation from further patients and will be reported subsequently. Nevertheless, the activity did seem to be consistent with a role of NA and GPi in the disorder.

**Figure 2:**
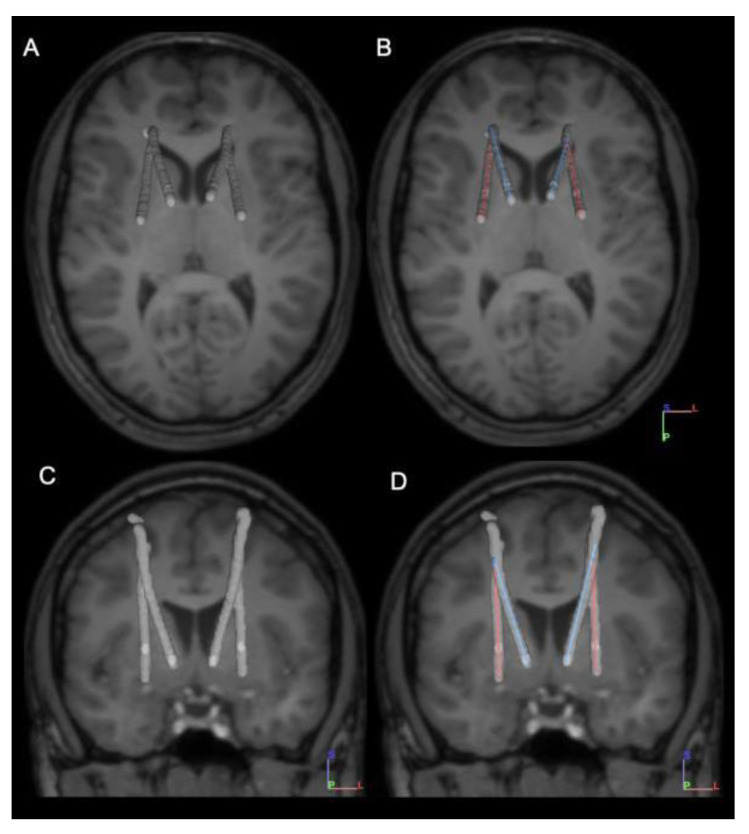
Axial (**A**,**B**) and coronal (**C**,**D**) views of the postoperative CT overlaid on the preoperative MRI, showing the lead locations for the Medtronic 3387 leads.

Based on the results of testing in the NMU, and in conjunction with the patient and his family, the clinical care team decided to implant permanent electrodes in bilateral Gpi posterior and NA. This decision was based on these targets showing maximal efficacy without side effects. Four permanent Medtronic 3387 DBS leads were implanted and connected to two Medtronic ActivaRC pulse generators (Medtronic Inc., Minneapolis, MN, USA) in the left and right chest. Targeting of the 3387 leads was based on the location of the effective Adtech depth electrodes, as identified on the initial postoperative CT scan. The AC-PC coordinates for the Medtronic leads are shown in Table 2. Standard stereotactic implantation procedures were followed, and both leads (NA and Gpi posterior) were implanted through a single burr-hole on each side of the head. Lead placement was confirmed with postoperative CT scan. Figure 2 depicts final permanent DBS lead placement location.

After implantation of permanent DBS leads and generators and activation of the pulse generators, the patient demonstrated substantial improvement in tics, with complete and persistent resolution of coprolalia. Initial setting of permanent electrodes were based on optimal settings noted in the NMU- with adjustments based on differing spacing on the Medtronic permanent electrode contacts from the macro-contacts on the sEEG leads as well as assessment of each contact at previous identified optimal frequency to find clinically effective contacts in each location. The patient was seen every 2–3 weeks during which voltage was gradually increased if indicated to sustain benefit. Adjustments in frequency and pulsewidth were made to assess for further benefit and limitation of side effects. Programming visits with a trained clinician (DF, JM, TS) continued every 2–6 weeks as indicated based on patient symptomatology. Intermittently during programming visits, blinded stimulation was performed to confirm results. The patient was noted to require a progressive increase in voltage in NA to maintain benefit. The Yale Global Tic Severity Scale (YGTSS) ratings were performed by a trained staff member (JM) and confirmed independently by the treating neurologist (TDS) (Table 3). The 15 month assessment was delayed from the standard 12 month assessment due to patient’s delay in returning to clinic as a result of the COVID-19 pandemic. The 57% reduction in tic severity exceeds the 25% threshold for positive response that has been proposed [16]. The patient was able to return to school, with significant reduction in overall tic severity and complete response of coprolalia that has been maintained up to two years post-surgery. Intermittent motor and simple verbal tics persist. The patient and parents stated strong satisfaction with the surgery, and there has been a self-reported reduction in anxiety and depressive symptoms.

## 4. Discussion

Severe medication-resistant Tourette syndrome presents a unique treatment challenge. Neurosurgical interventions have provided benefit to these patients but with incomplete responses, with improvement in YGTSS among the overall cohort ranging from 45% to 52.5% [1,10]. The efficacy of DBS is determined by many factors, target location is one of the most important. The targets for neurosurgical intervention vary greatly among individuals with Tourette syndrome. We suggest that placement of temporary electrodes allows for identifying optimal targets for each specific patient.

Although the NMU targeting procedure aided in the selection of successful targets for implantation of four permanent electrodes, the most surprising finding was the complete yet transient resolution of all symptoms following implantation of 12 electrodes. This result occurred prior to electrical stimulation through the temporary electrodes, suggesting the effect was due to a “micro-lesion” effect of the implantation itself, presumably caused by a localized inflammatory reaction around the electrodes that affects local brain activity. Since a total of 12 temporary leads were implanted, we cannot determine which lead(s) were responsible for the complete resolution. Subsequent electrical stimulation was based on the best individual stimulation targets, but electrical stimulation probably does not replicate the micro-lesion effect due to peri-electrode edema, so the results are not precisely comparable. Implantation of more than four leads (and two pulse generators) may increase surgical risks and postoperative complications, and thus we elected to implant only the four locations with greatest stimulation benefit, recognizing that this procedure was unlikely to completely replicate the results seen immediately following implantation of the MM16C leads. Stimulation testing suggests that GPi posterior, NA, and CM nuclei were likely to be important modulators in this patient. However, the “microlesion” effect is likely to affect the response to electrical stimulation, so electrical stimulation while hospitalized may be an incomplete predictor of final benefit. Furthermore, the full response to stimulation in Tourette syndrome is often not apparent for several days or weeks [17], and thus the short time during hospitalization may not predict the long-term outcome. Additionally, time constraints while the patient was hospitalized in the NMU limited the ability to explore all programming parameters on clinically effective leads.

The underlying cause of Tourette syndrome remains elusive, despite significant research and several important theoretical results [18,19,20,21]. Similarly, the mechanism of efficacy of DBS is poorly understood [22]. Tourette syndrome typically has a progressive response to DBS, suggesting that neuroplastic changes facilitate the response. In this case, neuroplastic changes may have been short lived since tics reoccur within minutes when DBS is turned off [23]. With DBS on, the decrease in tic severity reflected in the decrease in YGTSS score, has allowed the patient to re-enroll in school (Table 3). Tics continue, although frequency and intensity remain significantly decreased compared to pre-operative baseline.

Unfortunately, complete resolution comparable to the effect of the 12 temporary electrodes has not been achieved despite exhaustive testing of multiple programming parameters. This patient’s unique response raises further questions about the basis for the therapeutic effect of DBS in Tourette syndrome. The exact mechanism of action for DBS in Tourette syndrome and movement disorders remains to be elucidated [24]. It has previously been hypothesized that DBS inhibits abnormal nerve signaling through a lesion-like effect; however, more recent research suggests that the effect is more complex, involving intermittent excitatory and inhibitory effects that may correct abnormal neural circuit activity [25]. The current case suggests that Tourette syndrome may involve abnormal activity in a widespread network that requires intervention at multiple locations for resolution of symptoms.

The DBS procedure described here may allow for these issues to be investigated with a higher degree of safety than is possible with other approaches. In particular, unlike standard targeting procedures in which subjects may need to be awakened in the operating room to test for efficacy and side effects, in the NMU procedure, all surgery is done with patients under general endotracheal anesthesia, with testing performed subsequently while awake in a hospital bed. Furthermore, the NMU approach to testing provides parents and patients the opportunity to directly observe the effects of DBS and participate in the decision-making for target selection [12]. Finally, the NMU approach provides personalized therapy that may be required for Tourette syndrome patients due to their variable presentation.

## 5. Conclusions

In this case, we used sEEG depth electrodes for pre-implantation testing and target selection in treating a patient with Tourette syndrome. Tantalizingly, complete and immediate resolution of symptoms appears to be possible, but unfortunately the results in this case do not allow full determination of which combination of leads would have been most effective. Testing suggested that GPi posterior, NA, and CM parafascicular nuclei of thalamus are the most likely targets, and if symptoms continue to negatively impact this child’s school function, then consideration will be given to implantation of additional leads and pulse generators.

This case demonstrates the feasibility and importance of prolonged testing while children are awake and performing functional tasks. Further attention should be given to the optimal targets for patients with treatment-refractory Tourette syndrome undergoing DBS, including the need for multiple leads. For patients with Tourette syndrome who exhibit an incomplete response to single or double bilateral lead placement, additional lead placement may be needed. Further research and a larger case series are needed to confirm these results, to identify all the potential targets for DBS for the treatment of Tourette Syndrome, to identify patterns of abnormal electrical activity associated with tics, and to determine whether six or more leads may be required for complete resolution of symptoms. Consideration should be given to the design of new types of implanted leads and implanted pulse generators in order to accommodate the particular needs of patients with Tourette syndrome.

## Figures and Tables

**Table 1:** Target coordinates relative to AC-PC midpoint of temporary Adtech stereo EEG leads.

	AC-PC Coordinates	
Target	Left	Right
Vo STN	Lat: −13.5	Lat: 13.5
	AP: −2.0	AP: −2.0
	Vert: 1.0	Vert: 1.0
GPi Posterior	Lat: −21.5	Lat: 21.5
	AP: 4.0	AP: 4.0
	Vert: −3.0	Vert: −3.0
Gpi Anterior	Lat: −12.0	Lat: 12.0
	AP: 9.0	AP: 9.0
	Vert: −3.0	Vert: −3.0
CM Parafasciular nucleus	Lat: −5.0	Lat: 5.0
	AP: −4.0	AP: −4.0
	Vert: 0.0	Vert: 0.0
VIM Thalamus	Lat: −14.0	Lat: 14.0
	AP: −5.0	AP: −5.0
	Vert: 0.0	Vert: 0.0
NA	Lat: −9.3	Lat: 9.0
	AP: 13.93	AP: 14.68
	Vert: −0.87	Vert: −1.44

**Table 2:** Target coordinates relative to AC-PC midpoint of permanent Medtronic 3387 leads.

	AC-PC Coordinates	
Target	Left	Right
NA	Lat: −8.68	Lat: 9.71
	A-P: 13.62	AP: 13.18
	Vert: −3.37	Ver: −4.58
Gpi Posterior	Lat: −20.65	Lat: 22.9
	A-P:4.92	AP: 2.64
	Vert: −5.58	Vert: −6.58

**Table 3:** Patient’s YGTSS scores over the course of treatment. Stimulation parameters at time of each assessment noted below.

Number of Leads in Place		Stimulation YGTSS Score	
Initial presentation	None	N/A	100
Temporary electrode implantation	12	none	0
3 months after permanent lead implantation	4	+ ^1^	75
15 months after permanent lead implantation	4	+ ^2^	43

^1^ Left NA contacts 1+2+3-stimulation 0.5v, 200 μs, 225 Hz; Right NA contacts 9+10+11-stimulation 0.5v, 200 μs, 225 Hz; Left GPi contacts 0+1+2-3-stimulation 1v, 150 μs, 185 Hz; Right GPi contacts 8+9+10-11-stimulation 1v, 150 μs, 185 Hz. ^2^ Left NA contacts 1-2-3+Stimulation 6v, 120 μs, 240 Hz; Right NA contacts 9-10-11+Stimulation 6v, 120 μs, 240 Hz; Left GPi contacts 0+1+2-3-stimulation 1.5v, 180 μs, 60 Hz; Right GPi contacts 8+9+10-11-stimulation 1.5v, 180 μs, 60 Hz. GPi stimulation set to cycling at 5 min stimulation on: 1 min stimulation off. Stimulation parameters are based on Medtronic 3387 lead programming nomenclature in which contact 0 is the most distal contact on the left electrode and contact 8 is the most distal contact on the right electrode.

## Data Availability

The data presented in this study are available on request from the corresponding author. The data are not publicly available due to patient privacy.
